# Shift Work and Occupational Stress in Police Officers

**DOI:** 10.1016/j.shaw.2014.10.001

**Published:** 2014-10-20

**Authors:** Claudia C. Ma, Michael E. Andrew, Desta Fekedulegn, Ja K. Gu, Tara A. Hartley, Luenda E. Charles, John M. Violanti, Cecil M. Burchfiel

**Affiliations:** 1 Biostatistics and Epidemiology Branch, Health Effects Laboratory Division, National Institute for Occupational Safety and Health, Morgantown, WV, USA; 2 Department of Epidemiology and Environmental Health, School of Public Health and Health Professions, The State University of New York at Buffalo, NY, USA

**Keywords:** occupational stress, police officer, shift work

## Abstract

**Background:**

Shift work has been associated with occupational stress in health providers and in those working in some industrial companies. The association is not well established in the law enforcement workforce. Our objective was to examine the association between shift work and police work-related stress.

**Methods:**

The number of stressful events that occurred in the previous month and year was obtained using the Spielberger Police Stress Survey among 365 police officers aged 27–66 years. Work hours were derived from daily payroll records. A dominant shift (day, afternoon, or night) was defined for each participant as the shift with the largest percentage of total time a participant worked (starting time from 4:00 AM to 11:59 AM, from 12 PM to 7:59 PM, and from 8:00 PM to 3:59 AM for day, afternoon, and night shift, respectively) in the previous month or year. Analysis of variance and covariance were used to examine the number of total and subscale (administrative/professional pressure, physical/psychological danger, or organizational support) stressful events across the shift.

**Results:**

During the previous month and year, officers working the afternoon and night shifts reported more stressful events than day shift officers for total stress, administrative/professional pressure, and physical/psychological danger (*p* < 0.05). These differences were independent of age, sex, race/ethnicity, and police rank. The frequency of these stressful events did not differ significantly between officers working the afternoon and night shifts.

**Conclusion:**

Non–day shift workers may be exposed to more stressful events in this cohort. Interventions to reduce or manage police stress that are tailored by shift may be considered.

## 1 Introduction

Shift work is common in many occupations, including those of police officers and other emergency responders. There is increasing evidence that shift work is associated with cardiovascular disorders, including myocardial infarction and ischemic stroke [1–4]. Numerous underlying mechanisms have been proposed to explain causal relationships between shift work and cardiovascular disease (CVD) [4–8]. Shift work may be directly related to increased risk for CVD due to circadian disturbance or misalignment between work time and an individual's internal circadian system [6], or indirectly related to CVD through several pathways such as work-related psychological and behavioral disorders [7].

A number of prospective studies have provided evidence that work stress is a risk factor for CVD [9–12]. Peter et al [13] found that work stress explained the effects of shift work on cardiovascular risk factors such as hypertension and atherogenic lipids. Later, Puttonen et al [7] proposed occupational stress as one of the pathways mediating the association between shift work and CVD. Although previous studies have compared differences in work stress between shift and non–shift workers [14–17], work stress as well as shift structure may vary by occupation. Police officers, in particular, appear to be exposed to a higher level of stressors [18,19] and exhibited greater risk of CVD [20] and a more adverse CVD risk factor profile [21].

Spielberger et al [22] classified police stressors into three categories: administrative and professional pressures, physical and psychological dangers, and lack of support within and outside the police organization. The frequency of these stressors may be dependent on many factors including their work shift, police rank, and duty location, and may fluctuate throughout the year.

Determining the frequency of each stressor encountered by shift working police officers may be of vital importance for developing effectively tailored stress management programs. Current programs provide approaches to dealing with stress in general or are targeted for acute incidents, i.e., critical incident management program. This approach may reduce the effectiveness of stress interventions for police officers [23]. The objectives of the present study were to investigate (1) whether the number of overall police work-related stressful events that occurred in the previous month and year varied across shift type, and (2) whether a similar pattern was apparent for subscales of police stress including administrative/professional pressure, physical/psychological danger, and lack of organizational support.

## 2 Materials and methods

### 2.1 Source of data

Data were collected between 2004 and 2009 as part of the Buffalo Cardio-Metabolic Occupational Police Stress study, a cross-sectional study with a primary focus on assessing whether police work-related stress was associated with subclinical CVD and metabolic outcomes. All 710 active duty police officers from the Buffalo, New York Police Department (New York, NY, USA) were invited to participate in the study. Pregnant officers (*n* = 2) were excluded from examinations. Of the 464 officers examined, 99 were excluded due to missing values for either shift work or police stress, generating a sample of 365 officers (265 men, 100 women). Each officer provided informed consent. The study was approved by the State University of New York at Buffalo Internal Review Board and the National Institute for Occupational Safety and Health Human Subjects Review Board.

### 2.2 Police stress assessment

Work stress was assessed using the Spielberger Police Stress Survey [22]. This survey is a 60-item self-report questionnaire designed to assess acute and chronic stress in police officers. For each of the 60 items, each participant was asked to estimate the frequency with which they experienced the event in the previous month and year. The frequency of each item was categorized as 0, 1, 2, 3–5, 6–9, 10 + times in the previous month and 0, 1, 2–5, 6–10, 11–24, and 25 + times in the previous year. Factor analysis of this survey was conducted previously by Spielberger et al [22] and generated three stress subscales based on the amount of variance accounted for by each factor. These three subscales were administrative and professional pressure (23 items), physical and psychological danger (24 items), and lack of support (13 items). The administrative/professional pressure captures the stress from the judicial system, public criticism toward police officers, performance and rewards imbalance, work and family conflicts, and low job control and decision making. Physical/psychological threats include dangerous situations such as responding to a felony in progress, exposure or witness of death and severe injury, high-speed chases, personal insults, and night shift work. Lack of support assesses the stress from strained relations with supervisors, coworkers, and non–police friends, inadequate support within the department, and political pressure within and outside the department.

### 2.3 Shift work information

Work information during the 1 year prior to the date of examination was derived from a database of payroll records for each participant. Officers were assigned to one of three fixed schedules implemented by the Police Department beginning in 1994; however, officers may also work other shifts as necessary, such as court appearance or being a substitute for a sick coworker during a scheduled day off. Day shift started between 4:00 AM and 11:59 AM (62.1% of the day shift workers started work at 7:00 AM, and 37.9% at 8:00 AM), the afternoon shift was between 12:00 PM and 7:59 PM (all afternoon shift workers started work at 4:00 PM), and the night shift was between 8:00 PM and 3:59 AM (all night shift workers started work at 8:00 PM). The typical work schedule included 4 days of work, 4 days off work, 4 days of work, and 3 days off work. The total number of work hours as well as hours worked on each shift during the year prior to the date of examination were calculated for each participant, and were standardized to a weekly basis. A dominant shift (day, afternoon, or night) was assigned to each participant based on the largest percent time a police officer worked on a specific shift schedule during the previous year. Similarly, a dominant shift in the previous month was also assigned to each participant according to the participant's largest percent time worked on a specific shift during the previous month.

### 2.4 Demographic information

Information on sex, age, marital status, race/ethnicity, rank, and workload were obtained from a self-report questionnaire. Workload was estimated by each officer based on the perception of the work activity levels in the district in which he/she served. The workload was considered high in an area that had many complaints and a high crime rate; it was moderate if there was a moderate number of complaints and an average crime rate; and it was low if the district was not busy, and had a low crime rate.

### 2.5 Statistical analysis

Descriptive statistics were used to characterize the study population. Due to the small number, Hispanics (*n* = 7) were combined with whites. Police rank was collapsed into two groups: Patrol officers versus others including higher ranks such as Sergeant, Lieutenant, Captain, and Detective. Where mean frequencies for the three subscales for police stressors were reported, the original categorical version for the number of times a participant experienced a particular event during the previous month and year was converted to a numeric version with 0, 1, 2, 4, 7.5, and 10 values for the previous month and 0, 1, 3.5, 8, 17.5, and 25 values for the previous year.

Analysis of variance (ANOVA) and analysis of covariance (ANCOVA) were used to compare the unadjusted and adjusted mean number of stressful events that occurred in both the previous month and year for officers who worked on different shifts. Variables that were associated with both shift work and police work stress including age, sex, race/ethnicity, and police rank, and were considered as potential confounders for adjustment. Multiple comparisons were also made for the frequency of stressors between afternoon shift versus day shift, night shift versus day shift, and afternoon shift versus night shift. All analyses were conducted using SAS 9.3 (SAS Institute, Cary, NC, USA).

## 3 Results

Participating officers had a mean age of 41.2 (SD = 6.6) years and were predominantly male (72.6%), white or Hispanic (79.7%), and patrol officers (72.9%) (Table 1). Police officers working night and afternoon shifts were significantly younger than those working the day shift (*p* < 0.05), and those working afternoon shift were most frequently changing shifts (*p* < 0.001). The distribution of sex, race/ethnicity, and police rank significantly differed across shift, with female and African American officers more likely to work day shifts, and patrol officers more likely to work night shifts. The distribution of work load differed significantly across shift (*p* < 0.008). There was 94% concordance between assignment of dominant shift during the previous month and year (weighted kappa = 0.905, 95% confidence interval: 0.863–0.947; data not shown).

Based on the total number of events in the previous month, each officer reported an average of more than three work-related stressful events per day and encountered administrative pressures and physical/psychological danger events more than once per day (number of total stressful events or subscale of stressful events in the previous month divided by 30 days) (Table 2). The unadjusted analyses showed that the mean number of total stressful events and subscale stressors such as administrative/professional pressure and physical/psychological danger occurring in the previous month differed significantly by shift (*p* < 0.001), with officers working the afternoon and night shifts reporting significantly more stressful events (*p* < 0.05) than those working on day shift (Table 2).

Among the total number of stressful events reported for the previous year, 60.5% was non–threat-related stressors [(164.5 + 66.7)/381.9 × 100%] (Table 3). Both unadjusted and multivariable adjusted associations of shift work with total stress, administrative/professional pressure, and physical/psychological threat were statistically significant when shift work and stress were derived using data from the previous year [*p* < 0.001 and *p* < 0.02, respectively (Table 3)].

## 4 Discussion

The current study showed that police officers working afternoon and night shifts reported a higher number of work-related stressors compared to those working on day shift. This pattern was similar when shift work and stress were derived using data from both the previous month and year. Adjustment for multiple potential confounders (age, sex, race/ethnicity, and rank) did not alter the observed associations appreciably.

In general, our findings are consistent with previous studies involving different populations [14,17,24–26]. A previous study conducted in a Danish general working population reported higher odds of work-related stress such as conflicts at work and low decision latitude among non–day workers compared with day workers [14]. Another epidemiological investigation was conducted among employees in the United Kingdom oil and gas industry, including those who worked on oil and gas offshore installations and onshore processing plants [24]. Researchers found that onshore shift workers had a significantly less favorable work environment including physical stressors, job demand, job control, skill discretion, supervisor support, and safety perceptions than day workers. Nabe-Nielsen et al [17] found that job demand, decision latitude, skill discretion, and supervisor support were lower in evening and night shift workers compared to day shift workers in a Danish eldercare sector. Previous studies also found that the nurses working non–day shifts have more work–family conflicts than those working day shifts [25,26].

The significant differences in the total number of stressful events that occurred in the previous month and year between non–day shift and day shift workers in the current study could be caused by differences in the intensity of work. The difference could also be caused by work content. The significant difference in the number of physical/psychological threats might illustrate that the job content was different across shift, and this difference might contribute to the difference reported for the administrative/professional pressure across shift. For example, the officers working on non–day shifts encountered significantly more physical/psychological threat-related events such as “responding to a felony in progress” and “dealing with family disputes and crisis situations”, and subsequently, they would also have a higher frequency of “court appearances on their day off or day following a night shift”, “experiencing negative attitudes toward them”, and “insufficient manpower to adequately handle a job”. Therefore, they might need a higher level of administrative and organizational support, and support from family, friends, and the community they served. However, these needs might not be seen from the administrators' perspectives [27]. The significantly fewer number of administrative and executive officers working on non-day shifts in the current study may provide evidence in support of this possibility. Furthermore, these unmet needs might be associated with the increased number of other stressors such as “fellow officers not doing their job” and “demands made by family for more time”. It would be worthwhile to investigate whether police officers working on non-day shifts encounter a higher number of stressful events in other agencies that have different supervising strategies and administration levels.

There may be an expectation that the main source of police work-related stress would be from physical/psychological threats. However, our findings showed that the police officers reported a higher frequency of administrative/professional pressure than physical/psychological threats. Most of the stressors police officers encountered in the previous month and year were not related to physical/psychological threats. This finding was consistent with a previous study showing that administrative/professional pressure was most frequently occurring among the three subscales of police stress [28]. Because physical/psychological threats are an inherent part of police work, there is relatively little that can be done to reduce their occurrence in the police workforce. However, it may be possible to reduce or eliminate many of the administrative and organizational stressors within a police department.

Our study differed from previous investigations in the way stress was measured. Boggild et al [14] used a questionnaire that was constructed from 23 questions to assess work-related psychosocial job demands, decision latitude, social support, conflicts, and job insecurity in a random sample drawn from the Central Population Register of Denmark [14]. Parkes [24] assessed perceived stress related to work environment in six dimensions taken from six questionnaires. In a health care sector, work demands, control, and support were measured using a different questionnaire [17]. Using different questionnaires in different studies may be reasonable because work stress may vary by occupational characteristics. However, whether these questionnaires were ideal to assess the specific work stress in the study population was not clear. The Spielberger Police Stress Survey questionnaire [22] used in our study was specifically designed for assessing police work-related occupational stress, and therefore, providing unique information involving police stress.

Self-reported shift work history was commonly used in previous epidemiological studies investigating the effect of shift work on physical and psychosocial outcomes. Recall bias has been frequently raised as a limitation in these studies [14,17,24–26]. However this study used the objective measurement of daily work history data, which minimizes recall bias and increases the precision of our results.

Self-reported information on police work-related stress might lead to recall bias. To our knowledge, there is no objective measurement available for assessing occupational stress. Therefore, recall bias regarding police work stress was unavoidable and unmeasurable. However, we were able to illustrate its presence to a certain degree by using the self-reported stressors in the previous month and year under an assumption that police officers were likely to have better recall of the most recent events, i.e., those occurring in the previous month compared to those occurring over the previous year. If the number of stressful events that were reported in the previous month is multiplied by 12 (to represent the past year), the product is much greater than the self-reported number of stressful events occurring in the previous year. This discrepancy could be caused by seasonal crime variation or recall bias. Seasonal crime variation was less likely to have an effect on the results, because the participants were randomly selected for stress screening and the examination dates covered several years from 2004 to 2009 and from spring to winter in each year. The observation of similar results when stress and shift work were assessed in the previous month and year suggests that the recall bias did not influence the association substantially.

Another limitation to consider is the generalization of these results. Stress may vary by police rank, department size, type of community served, and police organizational characteristics; therefore, these results may be generalized to other police departments of a similar size with similar characteristics of age, sex, race/ethnicity, and police rank that are serving similar communities.

Using the largest percentage of hours a participant worked on a particular shift to define the shift status for a participant is not the only approach in shift work research; therefore, we also used different percentage criteria, i.e., ≥60% (*n* = 364), ≥70% (*n* = 355), ≥80% (*n* = 349), ≥90% (*n* = 344), and 100% (*n* = 141) of hours a participant worked on a particular shift for each participant and we observed similar results (data not shown). This finding revealed that shift classification bias was not evident in the current study. In addition, the Spielberger Stress Survey questionnaire was developed based on police officers' perceptions of the intensity of specific stressors and the frequency of occurrence of these specific sources of stress in law enforcement. The 60-item questionnaire was chosen from 81 original items and was assumed to capture most sources of police work-related stressors, if not all. Although the characteristics of crimes as well as the societal environment may have changed over the past few decades since the questionnaire was developed, some stressors such as “Fellow officers not doing their jobs”, “Making critical on-the-spot decisions”, and “Responding to a felony in progress” were among the top five most frequently occurring in 1979 [22], and remain the top five most frequently occurring stressors in the current study population (data not shown). These results illustrate a degree of consistency over time for this questionnaire.

In summary, our results showed that exposures to police work-related stress were more prevalent among police officers working the afternoon or night shift than the day shift. Officers working these two shifts reported more total stressful events as well as more events in the subscales of administrative/organizational stress and physical/psychological danger, compared with those who worked the day shift. It is possible that work stress may act as a mediator of associations between shift work and certain health outcomes, yet this potential mediating effect needs to be tested in future prospective studies. The current study may provide support for stress reduction interventions that are tailored differently by shift in the law enforcement profession.

## Conflicts of interest

All authors declare no conflicts of interest. The findings and conclusions in this report are those of the authors and do not necessarily represent the views of the National Institute for Occupational Safety and Health.

## Figures and Tables

**Table 1:** Demographic characteristics by shift in the past year among 365 police officers

Variable	Total	Shift during the previous year			*p*†
		Day	Afternoon	Night	
	(*n* = 365)	(*n* = 180)	(*n* = 99)	(*n* = 86)	
Age (y)	41.2 (6.6)	43.2 (6.2)	40.1 (6.0)∗	38.3 (6.8)∗	<0.001
Number of shift changes‡	4.1 (5.4)	2.8 (5.4)	6.8 (5.3)∗	3.8 (4.6)	<0.001
Total work hours per week	29.7 (4.9)	29.8 (4.7)	29.6 (5.4)	29.9 (4.7)	0.917
Sex					
Male	72.6	58.3	90.9	81.4	<0.001
Female	27.4	41.7	9.1	18.6	
Marital status					
Single	12.1	11.4	8.0	19.2	0.195
Married	73.8	74.6	79.0	65.4	
Divorced	14.1	14.1	13.0	15.4	
Race/ethnicity					
White/Hispanic	79.7	71.6	91.8	82.6	<0.001
African American	20.3	28.4	8.3	17.4	
Rank					
Patrol officer	72.9	66.1	75.5	83.7	0.008
Sergeant/lieutenant/captain/detective/executive	27.2	33.9	24.5	16.3	
Workload					
High	63.0	58.1	62.9	73.3	0.008
Moderate	32.5	33.9	35.1	26.9	
Low	4.5	8.1	2.1	0.0	

Data are presented as mean (SD) for the continuous variables and *n* (%) for the categorical variables.

∗ *p* < 0.05 for the comparison between the afternoon and night shifts with day shift.

† Analysis of variance for continuous variables and Chi-square test or Fisher's exact test for categorical variables.

‡ Each officer was assigned to a fixed shift. Shift changes occurred under the circumstances of court appearance or being a substitute for a sick coworker during a scheduled day off.

**Table 2:** Frequency of police work-related stressful events in the past month across shift among 365 police officers

	Total no. of events (range)	Shift during the previous month			*p*†
		Day (*n* = 186)	Afternoon (*n* = 101)	Night (*n* = 78)	
Total stress	95.7 (0.0–346.5)				
Unadjusted		80.1 ± 55.3	113.0 ± 62.5∗	110.6 ± 62.4∗	<0.001
Age, sex, rank, and race/ethnicity adjusted		84.2 ± 4.7	110.5 ± 6.1∗	106.4 ± 6.8∗	0.002
Administrative/professional pressure	41.3 (0.0–177.0)				
Unadjusted		33.7 ± 25.6	50.2 ± 31.9∗	48.0 ± 34.0∗	<0.001
Age, sex, rank, and race/ethnicity adjusted		36.0 ± 2.3	48.4 ± 3.1∗	46.0 ± 3.4∗	0.005
Physical/psychological threat	36.9 (0.0–128.5)				
Unadjusted		29.0 ± 22.5	44.0 ± 24.7∗	46.7 ± 24.6∗	<0.001
Age, sex, rank, and race/ethnicity adjusted		30.9 ± 1.8	43.1 ± 2.4∗	44.2 ± 2.7∗	<0.001
Lack of support	17.5 (0.0–80.0)				
Unadjusted		17.4 ± 14.7	18.9 ± 15.4	15.8 ± 11.3	0.373
Age, sex, rank, and race/ethnicity adjusted		17.3 ± 1.2	19.0 ± 1.5	16.2 ± 1.7	0.438

Data are presented as mean ± SD for the unadjusted models and as mean and standard error for adjusted models.

∗ *p* < 0.05 for the comparison between the afternoon and night shifts with the day shift.

† From analysis of variance or covariance for any difference across shift.

**Table 3:** Frequency of police work-related stressful events in the past year across shift among 365 police officers

	Total no. of events (range)	Shift during the previous year			*p*†
		Day (*n* = 180)	Afternoon (*n* = 99)	Night (*n* = 86)	
Total stress	381.9 (0–1095.0)				
Unadjusted		329.0 ± 205.5	432.7 ± 198.0∗	434.2 ± 213.8∗	<0.001
Age, sex, rank, and race/ethnicity adjusted		348.2 ± 16.4	416.4 ± 21.3∗	419.6 ± 22.4∗	0.018
Administrative/professional pressure	164.5 (0–513.5)				
Unadjusted		139.7 ± 93.0	190.5 ± 95.0∗	186.5 ± 107.2∗	<0.001
Age, sex, rank, and race/ethnicity adjusted		148.6 ± 7.8	183.2 ± 10.1∗	179.6 ± 10.7∗	0.018
Physical/psychological threat	150.7 (0–388.5)				
Unadjusted		125.7 ± 84.3	169.1 ± 81.0∗	181.8 ± 79.1∗	<0.001
Age, sex, rank, and race/ethnicity adjusted		134.8 ± 6.5	161.9 ± 8.4∗	173.8 ± 8.9∗	0.002
Lack of support	66.7 (0–252.0)				
Unadjusted		63.5 ± 47.8	73.1 ± 48.8	66.0 ± 47.3	0.279
Age, sex, rank, and race/ethnicity adjusted		64.8 ± 3.9	71.3 ± 5.1	66.2 ± 5.4	0.609

Data are presented as mean ± SD for the unadjusted models and as mean and standard error for the adjusted models.

∗ *p* < 0.05 for the comparison between the afternoon and night shifts with the day shift.

† From analysis of variance or covariance for any difference across shift.
